# FGF2 is overexpressed in asthma and promotes airway inflammation through the FGFR/MAPK/NF-κB pathway in airway epithelial cells

**DOI:** 10.1186/s40779-022-00366-3

**Published:** 2022-01-29

**Authors:** Yuan-Yang Tan, Hui-Qin Zhou, Yu-Jing Lin, Liu-Tong Yi, Zhuang-Gui Chen, Qing-Dong Cao, Yan-Rong Guo, Zhao-Ni Wang, Shou-Deng Chen, Yang Li, De-Yun Wang, Yong-Kang Qiao, Yan Yan

**Affiliations:** 1 Guangdong Provincial Key Laboratory of Biomedical Imaging and Guangdong Provincial Engineering Research Center, The Fifth Affiliated Hospital, Sun Yat-Sen University, Zhuhai, 519000 Guangdong China; 2 Department of Pathology, The Fifth Affiliated Hospital, Sun Yat-Sen University, Zhuhai, 519000 Guangdong China; 3 Department of Pediatrics, The Third Affiliated Hospital, Sun Yat-Sen University, Guangzhou, 510630 China; 4 Department of Cardiothoracic Surgery, The Fifth Affiliated Hospital, Sun Yat-Sen University, Zhuhai, 519000 Guangdong China; 5 Department of Otolaryngology, Yong Loo Lin School of Medicine, National University Health System, National University of Singapore, Singapore, 119228 Singapore; 6 BGI-Shenzhen, Shenzhen, 518083 Guangdong China; 7 Central Laboratory, The Fifth Affiliated Hospital, Sun Yat-Sen University, Zhuhai, 519000 Guangdong China

**Keywords:** Airway epithelial cell, Airway inflammation, Asthma, Fibroblast growth factor 2 (FGF2), House dust mite chronic model

## Abstract

**Background:**

Airway inflammation is the core pathological process of asthma, with the key inflammatory regulators incompletely defined. Recently, fibroblast growth factor 2 (FGF2) has been reported to be an inflammatory regulator; however, its role in asthma remains elusive. This study aimed to investigate the immunomodulatory role of FGF2 in asthma.

**Methods:**

First, FGF2 expression was characterised in clinical asthma samples and the house dust mite (HDM)-induced mouse chronic asthma model. Second, recombinant mouse FGF2 (rm-FGF2) protein was intranasally delivered to determine the effect of FGF2 on airway inflammatory cell infiltration. Third, human airway epithelium-derived A549 cells were stimulated with either HDM or recombinant human interleukin-1β (IL-1β) protein combined with or without recombinant human FGF2. IL-1β-induced IL-6 or IL-8 release levels were determined using enzyme-linked immunosorbent assay, and the involved signalling transduction was explored via Western blotting.

**Results:**

Compared with the control groups, the FGF2 protein levels were significantly upregulated in the bronchial epithelium and alveolar areas of clinical asthma samples (6.70 ± 1.79 vs. 16.32 ± 2.40, *P* = 0.0184; 11.20 ± 2.11 vs. 21.00 ± 3.00, *P* = 0.033, respectively) and HDM-induced asthmatic mouse lung lysates (1.00 ± 0.15 vs. 5.14 ± 0.42, *P* < 0.001). Moreover, FGF2 protein abundance was positively correlated with serum total and anti-HDM IgE levels in the HDM-induced chronic asthma model (*R*^2^ = 0.857 and 0.783, *P* = 0.0008 and 0.0043, respectively). Elevated FGF2 protein was mainly expressed in asthmatic bronchial epithelium and alveolar areas and partly co-localised with infiltrated inflammatory cell populations in HDM-induced asthmatic mice. More importantly, intranasal instillation of rm-FGF2 aggravated airway inflammatory cell infiltration (2.45 ± 0.09 vs. 2.88 ± 0.14, *P* = 0.0288) and recruited more subepithelial neutrophils after HDM challenge [(110.20 ± 29.43) cells/mm^2^ vs. (238.10 ± 42.77) cells/mm^2^, *P* = 0.0392] without affecting serum IgE levels and Th2 cytokine transcription. In A549 cells, FGF2 was upregulated through HDM stimulation and promoted IL-1β-induced IL-6 or IL-8 release levels (up to 1.41 ± 0.12- or 1.44 ± 0.14-fold change vs*.* IL-1β alone groups, *P* = 0.001 or 0.0344, respectively). The pro-inflammatory effect of FGF2 is likely mediated through the fibroblast growth factor receptor (FGFR)/mitogen-activated protein kinase (MAPK)/nuclear factor kappa B (NF-κB) pathway.

**Conclusion:**

Our findings suggest that FGF2 is a potential inflammatory modulator in asthma, which can be induced by HDM and acts through the FGFR/MAPK/NF-κB pathway in the airway epithelial cells.

**Supplementary Information:**

The online version contains supplementary material available at 10.1186/s40779-022-00366-3.

## Background

Asthma is one of the most common chronic inflammatory diseases, with 339 million people suffering and 420,000 deaths attributed to the disease each year [1]. Among the main features of asthma, chronic airway inflammation is the core of asthma’s pathophysiology, leading to airway dysfunction and irreversible airway wall remodelling, and thus is the primary target for asthma treatments [2]. However, currently available anti-inflammatory drugs are not always effective. This may be due to the heterogeneity of asthma endotypes, in which the predominant inflammatory cell types are different and exhibit distinct drug sensitivities. Equally important is the participation of airway structural cells, that is, the airway epithelial cells (AECs) and airway smooth muscle cells (ASMs), which were initially considered passive responders, but were later considered to be the key immunoregulatory cells in asthma [3]. These resident airway cells are the targets and sources of various inflammatory mediators and crosstalk with immune cells in the perpetuation of chronic inflammation via autocrine or paracrine pathways. Immunomodulatory networks involving airway resident cells are still not fully understood, and they are rarely designed as therapeutic targets. In this study, we provide evidence on the upregulation of fibroblast growth factor 2 (FGF2), also known as basic fibroblast growth factor (bFGF), in both airway-resident cells and inflammatory cell populations in patients with asthma, which may shed light on the mechanisms of airway epithelium-driven inflammation.

FGF2 belongs to the larger FGF family, and four high-affinity receptor tyrosine kinases have been identified as FGF receptors (FGFRs), including FGFR1 to FGFR4 [4]. One clinical study has shown that the level of FGF2 in the bronchoalveolar lavage fluid (BALF) collected from asthmatic patients is significantly higher than that in healthy subjects and can be further induced after allergen exposure [5]. Furthermore, FGF2 levels in sputum corrected by lung function are biomarkers of asthma severity [6], indicating that FGF2 may be involved in the pathogenesis of asthma. Nevertheless, the expression pattern of FGF2, that is, the expression levels and cellular locations of the FGF2 protein in asthmatic lungs, has not been fully investigated, as well as the role of FGF2 in modulating chronic airway inflammation in patients with asthma. In chronic inflammatory diseases such as atherosclerosis and chronic arthritis, FGF2 functions as a pro-inflammatory factor by activating macrophages or cooperating with IL-17 [7, 8], whereas in influenza A virus (IAV) infection, FGF2 protects against acute lung injury by recruiting neutrophils [9]. However, exogenous FGF2 administration was reported to suppress airway inflammation in an ovalbumin (OVA)-induced acute mouse model [10]. Considering the possible role of FGF2 in airway epithelial function, which may be entangled with the immune system and cause persistent chronic inflammation, a more clinically relevant disease model is required to investigate the role of FGF2 in asthma [3]. Therefore, in this study, a chronic mouse asthma model induced by the house dust mite (HDM), one of the main allergens inducing allergic asthma [11], was applied to investigate the potential immunomodulatory role of FGF2 in asthma patients. Moreover, clinical asthmatic samples were collected to verify the clinical significance of our observations.

## Methods

### Subjects

We compared FGF2 expression levels in lung samples from five asthmatic and five non-asthmatic subjects. Lung samples were collected from either biopsies or lobectomies at the Fifth Affiliated Hospital, Sun Yat-sen University, from 2019 to 2021. The patients have been additionally diagnosed with lung adenocarcinoma (three asthma cases, aged 48–77 years, and five non-asthma cases, aged 45–58 years), pulmonary bullae (one asthma case, aged 18 years), or pulmonary nodules (one asthma case, aged 47 years). The clinical characteristics of these patients are outlined in Additional file 1: Table S1. To avoid observation bias, the lung samples included in this study were all collected from non-tumour or normal regions, which were at least 5 cm away from the tumour or bullous lesions. The clinical study was approved by the Ethics Committee of the Fifth Affiliated Hospital, Sun Yat-sen University (approval No. K233-1).

### HDM-induced mouse asthma model

**Fig. 1:**
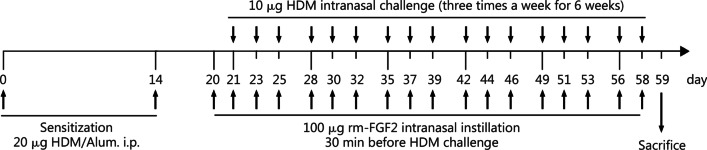
Protocol for HDM-sensitised and HDM-challenged chronic mouse model. HDM house dust mite, Alum aluminum hydroxide, i.p. intraperitoneal, rm-FGF2 recombinant mouse FGF2

BALB/c female mice (*n* = 34), aged 6 weeks, were purchased from Beijing Vital River Laboratory Animal Technology Co., Ltd. For the chronic asthma model, mice were divided into four groups: the saline group (as the normal control, *n* = 4), composed of unsensitised mice which receive i.p. injection and intranasal instillation of saline; the HDM group (*n* = 7); the HDM + rm-FGF2 group (*n* = 7); and the rm-FGF2 group (as rm-FGF2 control, *n* = 4), consisted of unsensitized mice which received intranasal instillation of rm-FGF2. The mice in groups HDM and the HDM + rm-FGF2 were sensitised through i.p. injection of 20 μg of HDM (XPB820D3A25, Greer Laboratories, Lenoir, North Carolina, USA) combined with 1 mg of aluminium hydroxide (A1577, Sigma Aldrich, Darmstadt, Germany) constituted in 0.1 ml of saline on days 0 and 14. The mice were further challenged with intranasal instillation of 10 μg of HDM in 30 μl of saline three times per week from weeks 3 to 8. In the HDM + rm-FGF2 group, 100 ng of recombinant mouse FGF2 (rm-FGF2; C044, NovoProtein, Jiangsu, China) in 30 μl saline was administered intranasally 30 min prior to the HDM challenge (Fig. 1). For the acute asthma model, mice were divided into four groups (*n* = 3): the saline group, the HDM group, the steroid treatment group (HDM + budesonide), and the solvent control group (0.1% DMSO was used as solvent for budesonide). The mice in the HDM and the steroid treatment groups were sensitised as the chronic asthma model sensitised procedure. The mice were further challenged with intranasal instillation of HDM (10 μg in 30 μl of saline) three times, with a 1-day interval each time. In the steroid treatment group, 0.5 mg/kg of budesonide (Hy-13580, MedChemExpress, Monmouth Junction, New Jersey, USA) was instilled intranasally 1 h prior to each HDM challenge, and 0.1% of DSMO was used as a negative control (Additional file 2: Fig. S1a). At 24 h after the final challenge, mice were anesthetised through the i.p. injection of 2,2,2-tribromoethanol (T48402, Sigma Aldrich), and cardiac puncture was performed to obtain the serum. The lung tissues were kept in liquid nitrogen, RNALater (R0901, Sigma Aldrich), fixed in 4% formalin, and then embedded in paraffin. The mice were housed in the animal unit at the Guangdong Provincial Key Laboratory of Biomedical Imaging. All animal experiments were performed in accordance with the guidelines of the Laboratory Animal Science Research Committee of the Fifth Affiliated Hospital, Sun Yat-sen University (approval No. 00119).

### Cell culture

The A549 cell line was obtained from ATCC and cultured in Dulbecco’s modified Eagle’s medium (DMEM; Gibco, Thermo Fisher Scientific, Waltham, Massachusetts, USA) supplemented with 10% foetal bovine serum. To verify whether FGF2 expression could be induced by HDM, A549 cells were starved for 24 h, the cells were stimulated with 10 μg/ml HDM, and cell lysates were collected at 0, 2, 4, 6, 8, and 12 h. To determine the effect of FGF2 on interleukin-1β (IL-1β)-induced IL-6 or IL-8 levels and explore the underlying mechanism, A549 cells were treated with 100 ng/ml recombinant human FGF2 (rh-FGF2; AF-100-18B, PeproTech, Rocky Hill, New Jersey, USA) 1 h prior to 1 ng/ml IL-1β (200-01B, PeproTech) stimulation. The supernatant was collected 48 h after stimulation for IL-6 and IL-8 release levels. In a parallel experimental group, 50 nmol/L of PD173074 (S1264, Selleckchem, Houston, Texas, USA), a specific FGFR inhibitor, was pre-added 1 h before the administration of 5 ng/ml rh-FGF2 combined with 1 ng/ml IL-1β to the cells, and the cell lysates were collected in 15 min for signal transduction analysis.

### Histopathological analysis

Mouse lung tissues embedded in paraffin were cut into 5-μm-thick sections and stained with haematoxylin and eosin (H&E; PH0516, Phygene, Fujian, China). The degree of inflammatory cell infiltration was scored according to the following histologic grading: 0, absence of peribronchial inflammatory cells; 1, scattered peribronchial inflammatory cells surrounding < 25% of the surrounding airways; 2, focal peribronchial inflammatory cells surrounding > 25%, but less than 100% of the bronchus; 3, one layer of inflammatory cells surrounding the bronchus; and 4, more than one layer of inflammatory cell infiltration surrounding the bronchus. For each mouse lung section, the inflammation score was calculated by adding the scores of all the airways 100–500 μm in diameter and dividing this score by the number of airways measured.

### Real-time quantitative RT-PCR (RT-qPCR)

The RNeasy RNA extraction kit (74106, Qiagen, Hilden, Germany) and TRIzol (15596018, Thermo Fisher Scientific) were used to extract total RNA from mouse lung tissues and cell cultures, respectively, according to the manufacturer’s instructions. Next, cDNA was synthesised using a reverse transcription kit (E047-01A, NovoProtein), and real-time quantitative-PCR (RT-qPCR) was performed using an SYBR Green-based qPCR kit (E096-01B, NovoProtein) and amplified on a CFX real-time PCR detection system (CFX96, Bio-Rad, Hercules, California, USA). The primer sequences used in this study are listed in Additional file 1: Table S2. The mRNA levels of the genes included were normalised to those of the housekeeping gene GAPDH (human) or Rpl13a (mouse) and depicted as 2^−ΔΔCt^.

### Enzyme-linked immunosorbent assay (ELISA) for mouse serum and cell supernatant

Total IgE and anti-HDM IgE levels in the serum were measured using BD OptEIA™ ELISA kits (555248, BD Biosciences, Franklin Lakes, New Jersey, USA) and mouse anti-HDM IgE ELISA kit (3037, Chondrex, Woodinville, Washington, USA), following the manufacturer’s instructions. The standard and duplicated samples were diluted 3000 times for total IgE detection and 25 times for anti-HDM IgE detection with diluted buffer, respectively. To measure IL-6 and IL-8 release levels in A549 cells, the human IL-6 ELISA kit or human IL-8 ELISA kit (R&D Systems, Minneapolis, Minnesota, USA) were used following the manufacturer’s protocols.

### Immunofluorescence staining for the lung sections

The slides were dried, de-waxed in xylene, and rehydrated in descending concentrations of ethanol to distilled water, followed by antigen retrieval in 10 mmol/L sodium citrate buffer (pH = 6) and heating for 20 min at 95 °C. After washing briefly in phosphate buffered saline (PBS), the sections were permeabilised with 0.3% Triton-X 100 for 20 min, and image iT-FX (I36933, Thermo Fisher Scientific) was added. The sections were then blocked in 5% goat serum (PH0424, Phygene) and 2% bovine serum albumin (BSA, NBS-BSA, Neobioscience, Guangdong, China) for 30 min. After washing in PBS, the human lung sections were incubated overnight at 4 °C with diluted antibodies against FGF2 (1:50 dilution, TD6038, Abmart, Shanghai, China) and pro-surfactant protein C (pro-SPC; 1:50 dilution, sc-518029, Santa Cruz, Dallas, Texas, USA). Mouse lung sections were incubated with diluted antibodies against FGF2 (1:100 dilution, sc-74412, Santa Cruz), Clara cell secretory protein (1:100 dilution, 07623, Sigma), pro-SPC (1:500 dilution, 3537117, Millipore, Darmstadt, Germany), or Ly-6G/C (1:50 dilution, sc-59338, Santa Cruz). The sections were then incubated with Alexa Fluor-labelled secondary antibody for 1 h at room temperature and stained with DAPI for 15 min. The slides were mounted with Prolong Gold Antifade (PH0429, Phygene), and the images were obtained using a laser confocal microscope (Zeiss, Oberkochen, Germany) or fluorescence microscope (Olympus, Tokyo, Japan).

### Western blotting

Mouse lung tissues or cultured A549 cells were lysed with RIPA buffer (89900, Thermo Fisher Scientific, USA) containing 1 × protease inhibitor and phosphatase inhibitor (P1045, Beyotime, China). Protein concentrations were measured using the bicinchoninic acid assay (PH0326, Phygene). Equal amounts of proteins (25 or 30 μg) were subjected to gel electrophoresis and transferred onto polyvinylidene fluoridemembranes. These membranes were then blotted with FGF2 (1:1000 dilution, sc-74412, Santa Cruz) or GAPDH (1:1000 dilution, 5174, Cell Signaling Technology, Danvers, Massachusetts, USA) for mouse samples and FGF2 (1:1000 dilution, 20102, Cell Signaling Technology), phospho-p38MAPK (Thr180/Tyr182; 4511, Cell Signaling Technology), phospho-ERK1/2 (Thr202/Tyr204; 4370, Cell Signaling Technology), p38MAPK (8690, Cell Signaling Technology), ERK1/2 (4695, Cell Signaling Technology), phospho-NF-κB p65 (Ser536; 3033, Cell Signaling Technology), phospho-IκBα (Ser32; 2859, Cell Signaling Technology), NF-κB p65 (8242, Cell Signaling Technology), or GAPDH (5174, Cell Signaling Technology) for A549 cell lysates. Subsequently, HRP-conjugated anti-rabbit (1:2000 dilution) or anti-mouse (1:3000 dilution) antibodies were used as secondary antibodies, and enhanced chemiluminescence (1705060, Bio-Rad) was conducted. Densitometry was performed using ImageJ (NIH, Bethesda, Maryland, USA) software.

### Statistical analysis

Statistical analysis was performed using unpaired Student’s *t*-test. The significance of the association between FGF2 and serum total IgE/anti-HDM IgE levels was evaluated using Pearson’s correlation. Zeiss ZEN microscope software was used to measure the fluorescence intensity of FGF2 staining in the airway epithelium. SPSS software (version 17.0; SPSS, Inc., Chicago, Illinois, USA) and GraphPad Prism 9.2.0 software (GraphPad Software Inc., San Diego, California, USA) were used to perform statistical analyses. Data were presented as the mean ± standard error of the mean (SEM) or *n* (%), and *P* values < 0.05 were considered statistically significant.

## Results

### FGF2 is overexpressed in asthmatic bronchial epithelium and alveolar areas

**Fig. 2:**
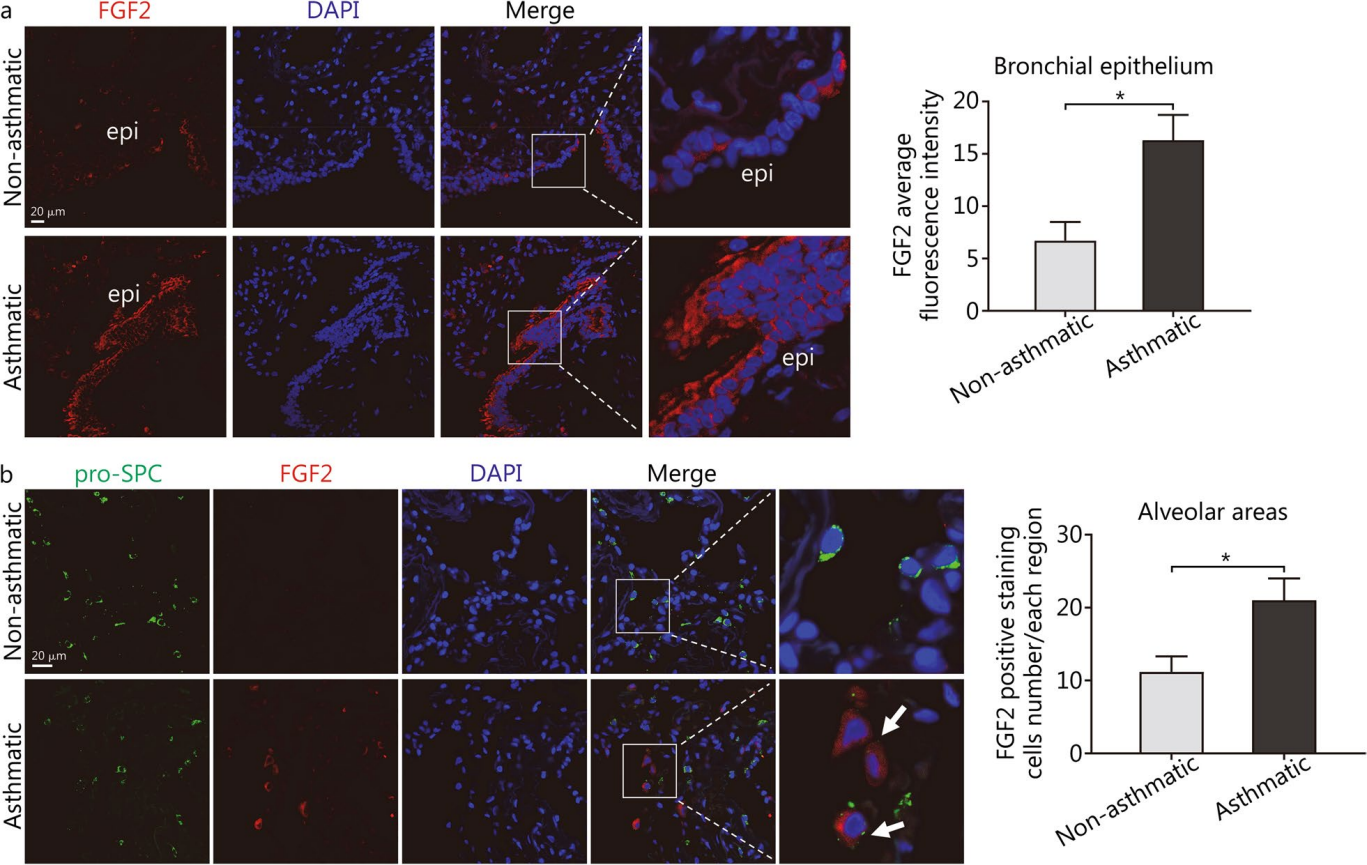
FGF2 was overexpressed in asthmatic lungs compared with non-asthmatic patients. **a** Representative immunofluorescence images for FGF2 in the lung sections to show the overexpression of FGF2 in asthmatic airway epithelium, compared with non-asthmatic patients (*n* = 5, scale bar = 20 μm). **b** Representative immunofluorescence images for FGF2 and pro-SPC in the lung sections to show FGF2 overexpression in asthmatic alveolar areas (*n* = 3–5, scale bar = 20 μm). Data are represented as mean ± standard error of the mean. ^*^P < 0.05. FGF2 fibroblast growth factor 2, epi epithelial, pro-SPC pro-surfactant protein C

To explore the expression pattern of FGF2 in asthmatic lungs, FGF2 expression in lung sections collected from asthmatic and non-asthmatic patients were compared by performing immunofluorescence staining. As shown in Fig. 2a, FGF2 protein staining intensity was significantly higher in the bronchial epithelium of asthmatic patients (6.70 ± 1.79 vs. 16.32 ± 2.40, *P* = 0.0184). Interestingly, a prominently higher ratio of FGF2-positive staining cells in the alveolar areas was also noticed (11.20 ± 2.11 vs. 21.00 ± 3.00, *P* = 0.033; Fig. 2b), which implicates the role of FGF2 in mediating the pathological changes in small airways in asthmatic patients.

### HDM-sensitised and chronically challenged mice exhibit significant chronic asthma hallmarks

**Fig. 3:**
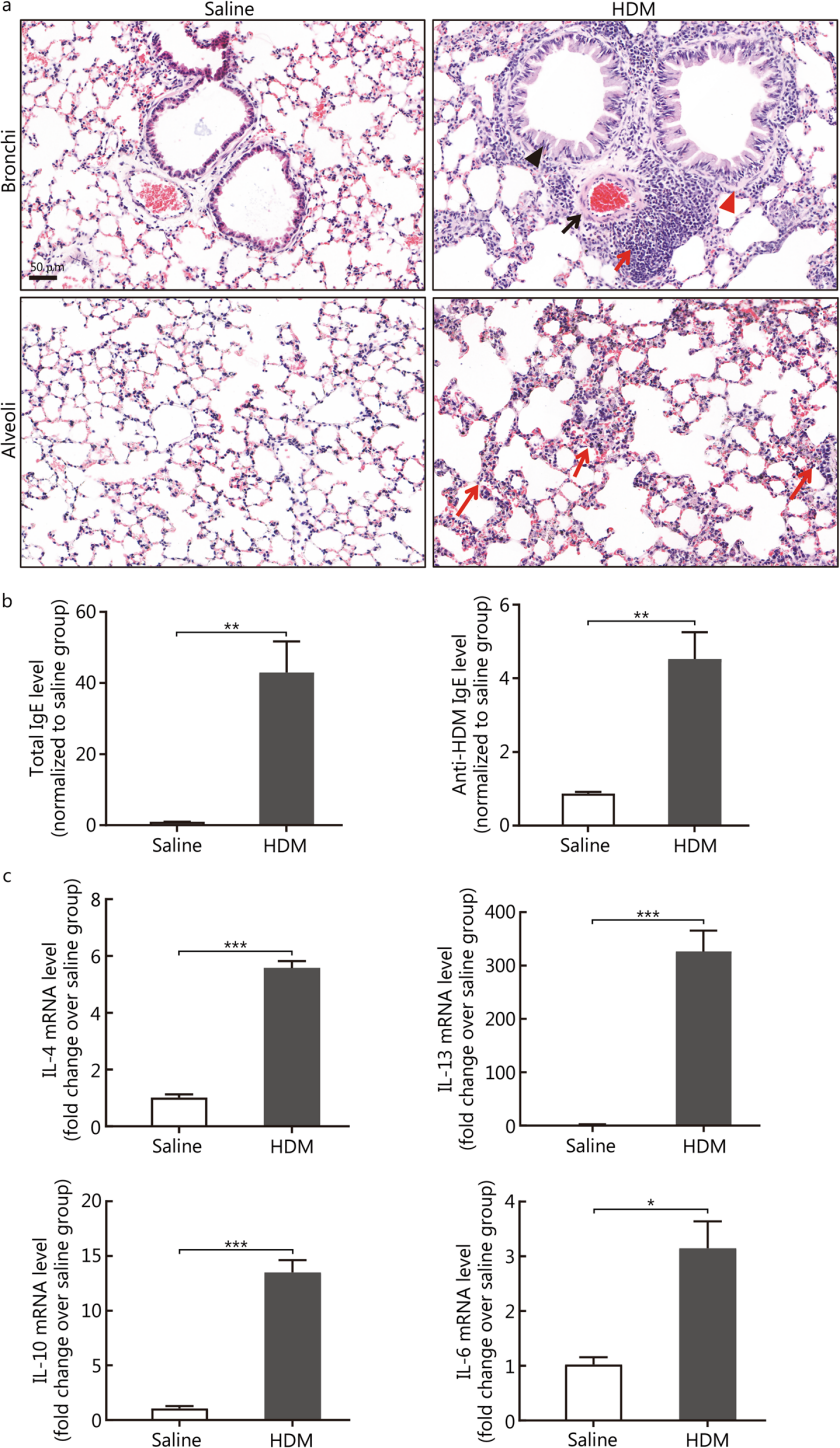
HDM-sensitised and HDM-challenged mice showed significant airway inflammatory cell infiltration, increased cytokine production, and increased serum levels for both total IgE and HDM-specific IgE. **a** Representative H&E images for the lung sections of the saline and HDM groups (scale bar = 50 μm). The black triangle indicates epithelial goblet cell hypertrophy, the black arrow indicates vascular smooth muscle thickening; the red triangle refers to airway smooth muscle thickening, and the red arrows indicate inflammation cell infiltration within the peribronchial, perivascular, and alveolar areas. **b** ELISA data showing the upregulation of the serum total IgE and anti-HDM specific IgE levels in the HDM group. **c** RT-qPCR results showing that the transcripts levels for asthma-related cytokines were significantly increased in the HDM group. The mRNA levels were normalised to the housekeeping gene, Rpl13a. Data are represented as mean ± standard error of the mean, *n* = 4 or 7. ^*^P < 0.05, ^**^P < 0.01, and ^***^P < 0.001 (through Student’s *t*-test). ELISA enzyme-linked immunosorbent assay, HDM house dust mite, IL interleukin

After HDM sensitisation and challenge, HDM-induced chronic asthmatic mice exhibited significant inflammatory cell infiltration around the vessels and airways, including the bronchi and alveoli, as well as structural changes, such as goblet cell hyperplasia/metaplasia and airway or vascular smooth muscle thickening in the lung sections (Fig. 3a). Additionally, total IgE and anti-HDM IgE levels in the serum significantly increased in the HDM group (1.00 ± 0.03 vs. 42.97 ± 8.74, *P* = 0.0063; 0.87 ± 0.04 vs. 4.52 ± 0.73, *P* = 0.0049, respectively; Fig. 3b), coupled with the elevation of the transcripts of asthma-related cytokines, including IL-4, IL-13, IL-10, and IL-6 (1.01 ± 0.11 vs. 5.58 ± 0.24, *P* < 0.001; 1.17 ± 0.32 vs. 326.40 ± 39.29, *P* < 0.001; 1.07 ± 0.23 vs. 13.5 ± 1.12, *P* < 0.001; 1.02 ± 0.13 vs. 3.15 ± 0.49, *P* = 0.0117, respectively; Fig. 3c), which represent a typical phenotype for allergic asthma.

### FGF2 protein abundance is upregulated in the HDM-induced mouse asthma model, positively correlated with serum IgE levels, and unaffected by budesonide administration

**Fig. 4:**
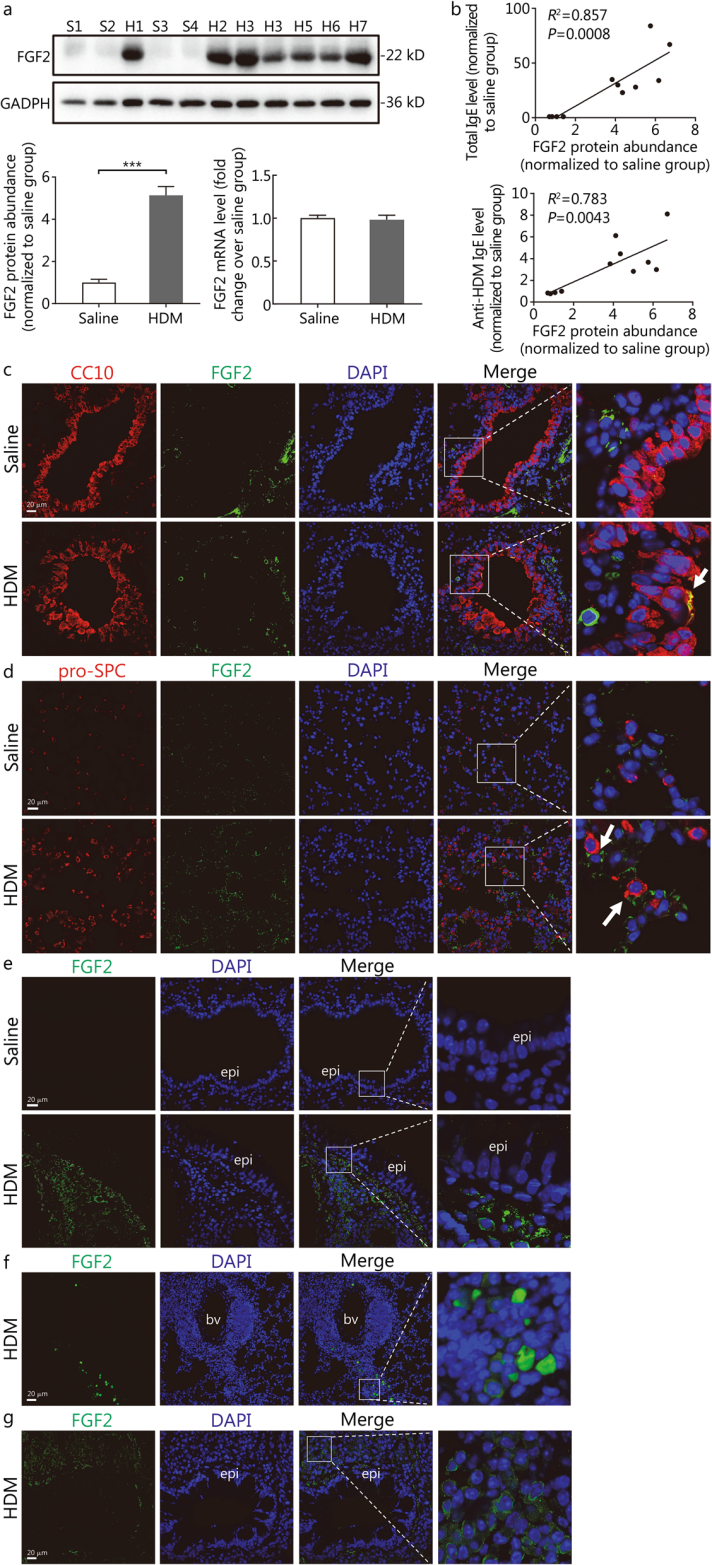
FGF2 protein abundance was higher in the HDM-treated mouse lungs and positively correlated with serum levels for total IgE and anti-HDM specific IgE. **a** Western blotting and RT-qPCR analysis for fibroblast growth factor 2 (FGF2) expression levels in the mouse model. GAPDH is used as a loading control. Data are represented as mean ± standard error of the mean (^***^P < 0.001) through Student’s *t-*test. **b** Correlation between FGF2 protein abundance and total IgE or anti-HDM-specific IgE levels in the serum. The *R*^2^ and *P* values are determined through Pearson’s correlation analysis. Representative confocal images of the mouse lung section for FGF2 staining around the **c** bronchi and **d** alveolar areas (scale bar = 20 μm). White arrows indicate FGF2-positive staining. Representative immunofluorescence images for FGF2 deposition in **e** the subepithelial basement membrane, **f** perivascular inflammatory cells, and **g** peribronchial inflammatory cell populations in HDM-treated mouse lungs (scale bar = 20 μm). S saline, H house dust mite (HDM), CC10 Clara cell secretory protein, pro-SPC pro-surfactant protein C, epi epithelium, bv blood vessel

To verify whether FGF2 is consistently overexpressed in the murine asthma model, both the protein abundance and transcription levels of FGF2 between the HDM and saline groups were compared. As shown in Fig. 4a, compared with the saline group, FGF2 protein abundance (the 22-kD isoform) was significantly increased in the HDM group (1.00 ± 0.15 vs. 5.14 ± 0.42, *P* < 0.001), but no statistical difference in the transcription level was observed (1.00 ± 0.03 vs. 0.98 ± 0.05, *P* = 0.798). Interestingly, FGF2 protein abundance was positively correlated with total IgE and anti-HDM IgE levels in the serum, suggesting an association between FGF2 expression and allergen stimulation (*R*^2^ = 0.857 and 0.783, *P* = 0.0008 and 0.0043, respectively; Fig. 4b). To explore whether the upregulation of FGF2 expression could be inhibited by steroid treatment, an HDM-induced acute mouse asthma model was applied, and budesonide was administered as described in Additional file 2: Fig. S1a. As expected, an upregulation of FGF2 protein abundance (the 22-kD isoform) was consistently observed in this acute asthma model (1.00 ± 0.14 vs. 5.54 ± 0.55, *P* = 0.0013; Additional file 2: Fig. S1c). Although budesonide effectively suppressed airway inflammatory cell infiltration in HDM-sensitised and HDM-challenged mouse lungs, the FGF2 protein abundance was not affected, since a significant difference between the HDM + budesonide and HDM + DMSO control groups was not observed (4.08 ± 0.56 vs. 3.55 ± 0.44, *P* = 0.4996; Additional file 2: Fig. S1b-c).

### FGF2 is overexpressed in both bronchial and alveolar areas in the chronic asthma model

To further determine the cell types that overexpress FGF2 in patients with asthma, immunofluorescence staining for FGF2 was performed in the lung sections of the mouse chronic asthma model. As indicated in Fig. 4c, after HDM sensitisation and chronic challenge to the mice, the FGF2 protein levels were upregulated in bronchial epithelial cells, as indicated by the co-staining of FGF2 and a marker of AECs, CC10. Except for the peribronchial regions, a significant elevation of FGF2 protein abundance was found in the lung parenchyma, expressed around the alveolar type II epithelial cells (AEC II, pro-SPC positive, Fig. 4d). These phenomena are consistent with our observations of the clinical samples, as shown in Fig. 2. Moreover, significant deposition of the FGF2 protein was also observed in the basement membrane of the bronchi in the HDM mice, whereas it was negligible in the saline group (Fig. 4e). Moreover, co-localisation of the FGF2 protein and inflammatory cells around the vessels and bronchi was found (Fig. 4f–g), which might imply a direct role of FGF2 in immune cell populations.

### rm-FGF2 promotes airway inflammatory cell infiltration and recruits subepithelial neutrophils in the chronic mouse model

**Fig. 5:**
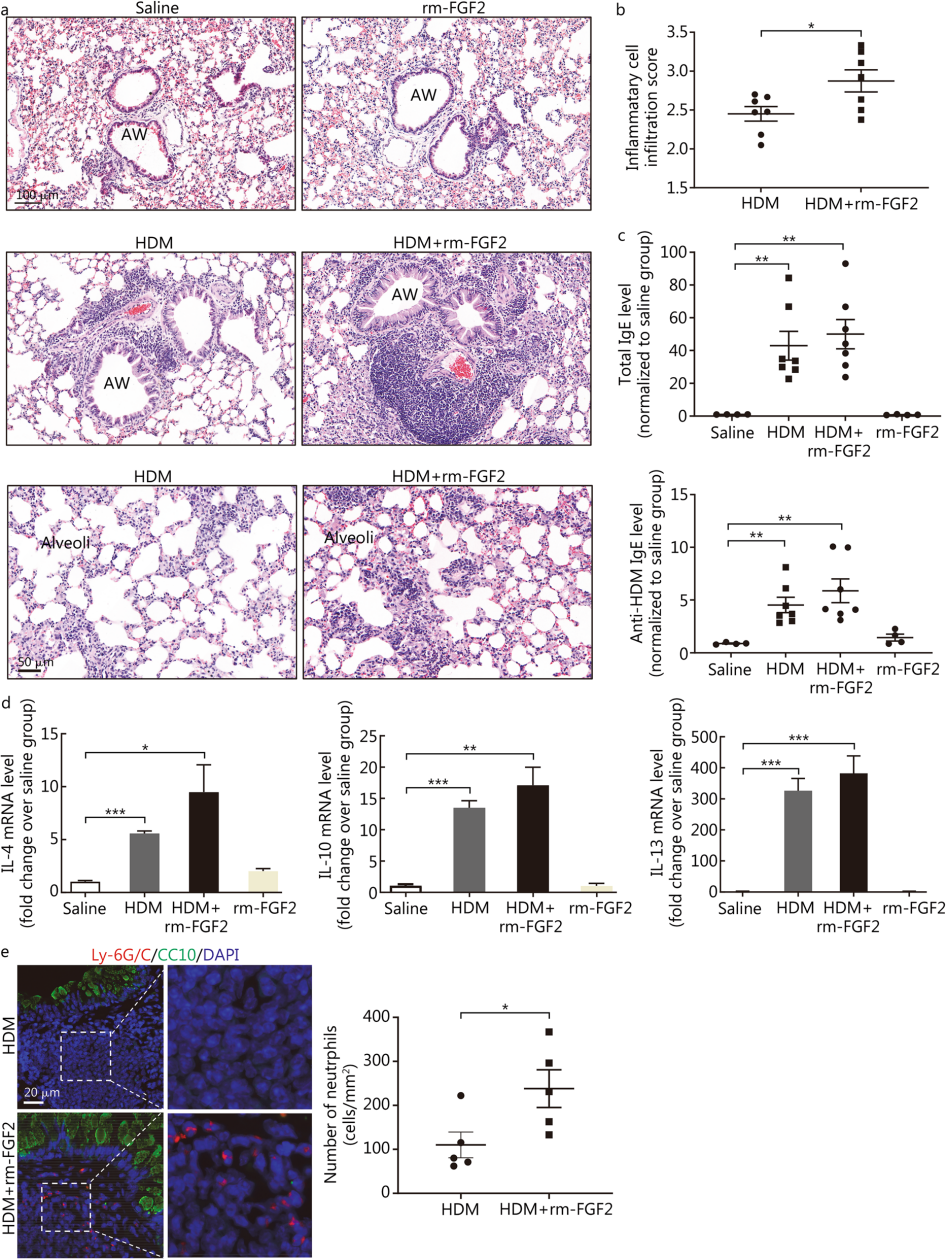
rm-FGF2 intranasal instillation promoted HDM-induced airway inflammatory cell infiltration and recruited subepithelial neutrophils in the mouse model. **a** Representative H&E staining images for the lung sections. Scale bar for the bronchial airway images = 100 μm and for the alveolar area images = 50 μm. **b** Score of airway inflammatory cell infiltration levels between the HDM and HDM + rm-FGF2 groups (*n* = 7). **c** ELISA results showing the total IgE and anti-HDM-specific IgE levels in the sera among experimental groups (*n* = 4 or 7). **d** Analyses of the transcription levels for IL-4, IL-10, and IL-13 genes in each group through RT-qPCR. mRNA levels were normalised to the housekeeping gene, Rpl13a. **e** Representative immunofluorescence images and statistical analysis showing that the number of subepithelial neutrophils was increased in the HDM + rm-FGF2 group compared with that in HDM alone group (*n* = 5, scale bar = 20 μm). Data are represented as mean ± standard error of the mean. ^*^P < 0.05, ^**^P < 0.01, and ^***^P < 0.001. AW airway, rm-FGF2 recombinant mouse FGF2 protein, HDM house dust mite

To ascertain whether FGF2 exerts an immunomodulatory effect on asthma pathogenesis, 100 ng of rm-FGF2 was administered through intranasal instillation half an hour prior to the HDM challenge three times per week for up to 6 weeks (Fig. 1), and the airway inflammatory cell infiltration levels were compared between the rm-FGF2-treated and control groups through H&E staining and histopathological analysis. Strikingly, the airway inflammatory cell infiltration scores were significantly higher in the HDM combined with the rm-FGF2 group, compared with that in the HDM alone group (2.45 ± 0.09 vs. 2.88 ± 0.14, *P* = 0.0288; Fig. 5a, b). To exclude the possibility that the elevated inflammatory cell infiltration scores in the rm-FGF2 combined with HDM group were due to a higher allergic reaction, serum total IgE and anti-HDM IgE levels were measured, and significantly higher serum IgE levels were found in the HDM groups, but no difference between the rm-FGF2 pre-treatment and HDM alone groups was noted (*P* > 0.05, Fig. 5c). Next, we sought to determine whether FGF2 administration amplifies the type 2 immune response by measuring Th2 cytokine transcripts. Unexpectedly, a significant upregulation of IL-4, IL-10, and IL-13 transcripts was not observed in the rm-FGF2 treatment group compared with that in the HDM group, although there is an increasing trend (9.49 ± 2.57 vs. 5.58 ± 0.24, 17.12 ± 2.86 vs. 13.5 ± 1.12, and 382.40 ± 55.95 vs. 326.40 ± 39.29 for IL-4, IL-10, and IL-13, respectively, *P* > 0.05, Fig. 5d). Asthma is highly heterogeneous, and neutrophilic inflammation contributes to a subgroup of Th2-low endotypes in patients with asthma [12]. Consequently, we further speculate that FGF2 might work through the neutrophils that are independent of Th2-driven airway inflammation. Indeed, by performing immunofluorescence staining for a specific neutrophil marker, Ly-6G/C, we found that more neutrophils were recruited into the subepithelial regions in the rm-FGF2 pre-treatment group [(110.20 ± 29.43) cells/mm^2^ vs. (238.10 ± 42.77) cells/mm^2^, *P* = 0.0392; Fig. 5e].

### FGF2 is induced through HDM stimulation in AECs

**Fig. 6:**
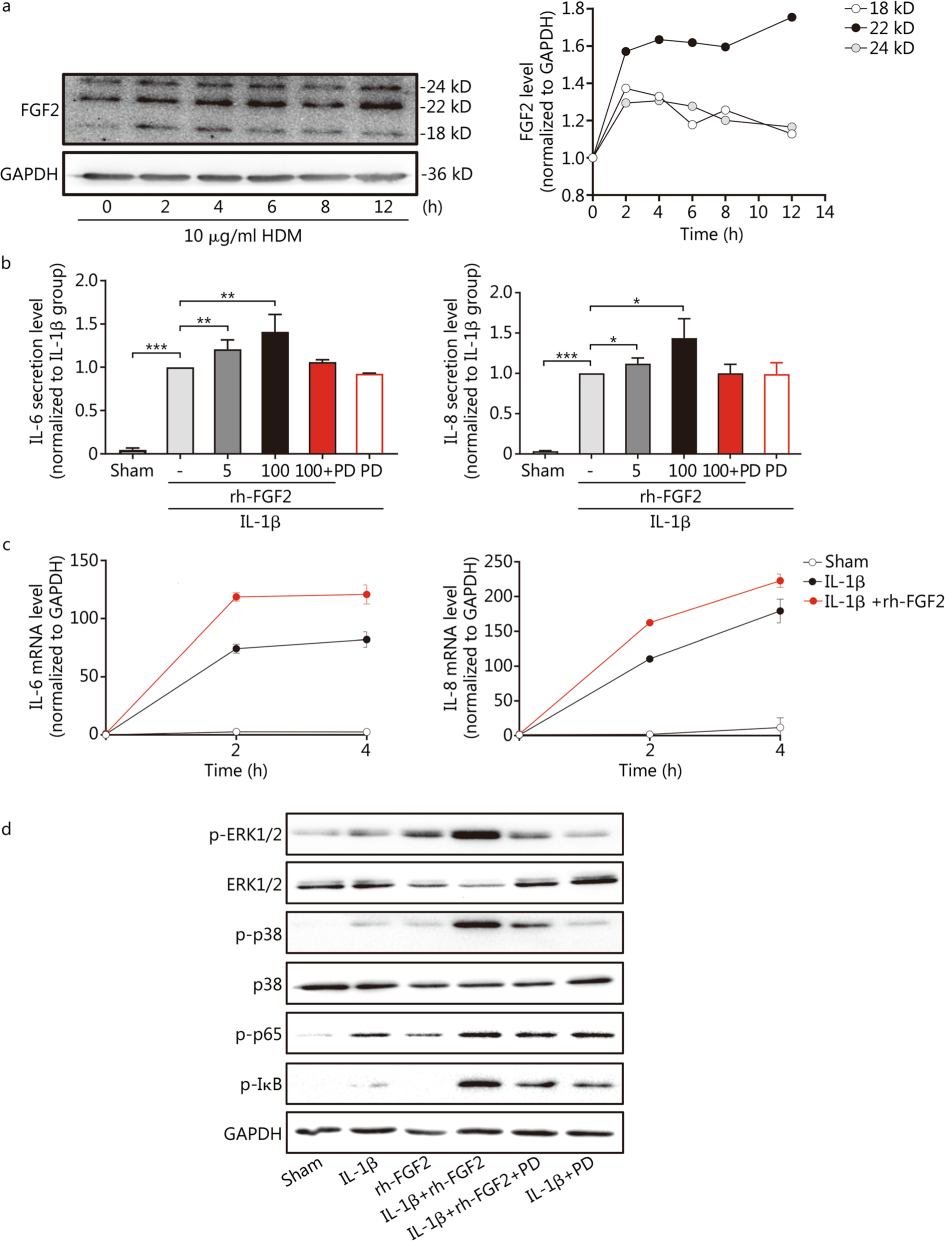
FGF2 was upregulated through HDM treatment and amplified the release levels of IL-6 and IL-8 induced by IL-1β in A549 cells via FGFR/MAPK/NF-κB signalling. **a** Western blotting analysis showing the upregulation of FGF2 protein abundance induced by HDM treatment in A549 cells at different time points. **b** IL-6 and IL-8 release levels were measured through ELISA 48 h post treatments (*n* = 3). Data are represented as mean ± standard error of the mean. **c** RT-qPCR results showing the IL-6 and IL-8 mRNA levels among treatment groups. mRNA levels were normalised to the housekeeping gene, GAPDH. **d** Representative Western blotting showing the protein levels of p-ERK1/2, total-ERK1/2, p-p38, total-p38, p-p65, and p-IκB in A549 cells 15 min post treatments. ^*^P < 0.05, ^**^P < 0.01, and ^***^P < 0.001. PD FGFR inhibitor PD173074, rh-FGF2 recombinant human FGF2, Sham negative control

Next, we determined whether aeroallergen stimulation is responsible for the upregulation of FGF2 in asthmatic epithelial cells. For this purpose, the AEC A549 line was used as a cell model, and the cells were treated with 10 μg/ml of HDM to determine FGF2 protein abundance at different time points through Western blotting. As a result, different FGF2 isoforms, including 18-, 22-, and 24-kD isoforms, were upregulated 2 h post-HDM stimulation (1.37-, 1.57-, and 1.29-fold changes, respectively), wherein the 22-kD isoform kept increasing up to 12 h post-stimulation (1.75-fold change), whereas the 18- and 24-kD isoforms gradually declined after 2 h (Fig. 6a).

### rh-FGF2 enhances IL-1β-induced IL-6 and IL-8 secretion in the A549 cell line

Based on the current evidence, we further hypothesised that FGF2 promotes airway epithelial-associated inflammation. Therefore, A549 cells were treated with the pro-inflammatory cytokine IL-1β, combined with or without the stimulation of rh-FGF2 at lower (5 ng/ml) and higher (100 ng/ml) doses, and IL-1β-induced IL-6 or IL-8 secretion levels were measured. We chose to examine IL-1β, IL-6, and IL-8, because these cytokines have been associated with neutrophilic asthma endotypes and secreted by AECs responding to environmental stimuli. By performing ELISA for the supernatant, rh-FGF2 significantly enhanced IL-1β-induced IL-6 or IL-8 release in a dose-dependent manner. A lower dosage of FGF2 (5 ng/ml) induced (1.21 ± 0.05)-fold changes in IL-6 or (1.12 ± 0.05)-fold changes in IL-8 secretion levels compared with that in the IL-1β alone groups (*P* = 0.0013 and 0.0486, respectively), whereas a higher dosage of FGF2 (100 ng/ml) induced (1.41 ± 0.12)- or (1.44 ± 0.14)-fold changes in IL-6 or IL-8 compared with that in the IL-1β alone groups (*P* = 0.001 and 0.0344, respectively) (Fig. 6b). Meanwhile, rh-FGF2 alone did not show any effect (data not shown). This is further supported by the RT-qPCR results showing that rh-FGF2 treatment significantly increased IL-1β-induced IL-6 or IL-8 transcript levels at 2 (1.60- and 1.47-fold changes, respectively) and 4 h (1.48- and 1.24-fold changes, respectively) post-stimulation (Fig. 6c). More importantly, the additional application of a specific FGFR inhibitor, PD173074, to the 100 ng/ml rh-FGF2 combined with the IL-1β groups decreased the IL-6 or IL-8 levels to levels comparable to those in the IL-1β alone group, indicating that the pro-inflammatory effect of FGF2 in AECs is FGFR-dependent (Fig. 6b).

### The pro-inflammatory effect of rh-FGF2 is partly mediated by the FGFR/MAPK/NF-κB pathway in AECs

Next, to explore the underlying signalling transduction pathway involved, cell lysates were collected 15 min post-stimulation in parallel settings. Both the total protein and phosphorylation levels of MAPKs (ERK and p38MAPK) and NF-κB were determined through normalisation to the housekeeping gene GAPDH. As shown in Fig. 6d, the phosphorylation levels of ERK1/2, p38 MAPK, p65, and IκB were significantly increased by IL-1β stimulation, indicating activation of MAPK/NF-κB signalling. Interestingly, while rh-FGF2 treatment alone did not affect NF-κB signalling, additional administration of rh-FGF2 to IL-1β greatly enhanced the levels of phospho-p65 and phospho-IκB. This was accompanied by a prominent upregulation of MAPK signalling, indicating that the presence of rh-FGF2 promotes crosstalk between MAPKs and NF-κB pathways, which contributes to increased cytokine production. More importantly, pre-treatment with PD173074, a specific FGFR inhibitor, significantly diminished the effect of rh-FGF2 on promoting MAPK and NF-κB activation after IL-1β treatment, implicating the involvement of an FGFR-dependent pathway (Fig. 6d).

## Discussion

This study revealed that FGF2 expression was consistently upregulated in clinical asthma samples and in HDM-induced asthmatic mouse lungs. Although the characterisation of FGF2 expression in asthma samples provides first-hand clues for the potential role of FGF2 in disease conditions, it has not been systemically studied prior to this study. It was demonstrated that FGF2 was predominantly expressed within the bronchi and equally prominent in alveolar areas in asthma groups. FGF2 elevation within bronchial areas is not surprising, considering the potential of FGF2 to be a pro-fibrotic factor that plays a role in subepithelial fibrosis in patients with asthma [13, 14]. The finding that FGF2 is overexpressed in bronchial epithelial cells is consistent with the findings of Shute et al.’s study [15], which indicates the potential of FGF2 in regulating airway epithelial dysfunction. In contrast, the overexpression of FGF2 protein in asthmatic alveolar areas has not been reported, and the underlying mechanisms are unclear. Compared to the large airways, the significance of alveolar pathological changes in patients with asthma, including alveolar infiltration of inflammatory cells that differ from the larger airways [16], alveolar fibrosis characterised by the accumulation of myofibroblasts [17], and small airway obstruction [18], has only been appreciated in recent years, and the underlying mechanisms remain poorly understood. In the clinical samples, an increase in FGF2-positive cells was observed, including not only AEC II (pro-SPC-positive cells), but also other cell populations that have not been characterised in the current study. These cell types could be fibroblasts or immune cells, which have been demonstrated to express FGF2 through the stimulation of pro-fibrotic factors or protease enzymes, such as TGF-β [19] or neutrophil elastase [20]. Further investigations of FGF2 in alveolar cells might shed light on the potential role of FGF2 signalling in mediating distal airway pathological changes in asthma.

Interestingly, our study in the HDM-induced asthma model suggests that FGF2 protein expressed in alveolar areas is mainly extracellular, which might be attributed to different FGF2 isoforms expressed in peri-bronchial and alveolar areas. In this context, both low-molecular-weight (LMW, 18-kD) and high-molecular-weight (HMW, 22-, 22.5-, 24-, and 34-kD) isoforms of FGF2 protein have been characterised so far, and these FGF2 isoforms have different subcellular localisations and functions [21]. LMW FGF2 is generally cytoplasmic and secreted, whereas HMW FGF2 is more likely to be located in the nucleus [22]. In our study, although the 22-kD FGF2 isoform is the major isoform that can be detected in the mouse study through Western blotting, both the LMW and HMW isoforms of FGF2 were upregulated through HDM stimulation in A549 cells. Future studies would be conducted to look into the regulatory mechanisms and the mode of action of these FGF2 isoforms in pulmonary cells.

Through the nasal instillation of rm-FGF2 to the HDM-induced chronic mouse asthma model, FGF2 promotes airway inflammatory cell infiltration in asthma patients, which is IgE- and Th2-independent. This indicates that additional mechanisms beyond allergic reactions and Th2 immune responses may account for the pro-inflammatory effect of FGF2 in asthma. Asthma is a highly heterogeneous disease that can be divided into several subtypes according to the distinct cellular and pathophysiological pathways involved [23, 24]. Among the asthma subtypes, eosinophilic and T2-high asthma is the most well-studied, which is featured by excessive eosinophil infiltration into the airways and a CD4^+^ T cell-driven type 2 immune response leading to a typical Th2 profile [25]. A sizable subgroup of asthma patients lacks a typical Th2 immune response and eosinophilic airway inflammation, named T2-low asthma [26]. The pathological and cellular mechanisms underlying T2-low asthma remain poorly understood. Among those T2-low asthma subtypes, a subgroup of patients exhibits a predominant neutrophilic phenotype associated with more severe later-onset asthma and worsening and persistent airway obstruction and tends to be resistant to corticosteroids [27, 28]. This asthma subtype has been recognised as neutrophilic asthma, which accounts for 15–25% of asthma cases [29]. The involved mechanisms include Th1- and Th17-mediated pathways [30], innate immune response [31], and viral infection, smoking [32]. In our study, FGF2 pre-treatment significantly induced the recruitment of subepithelial neutrophils, which provides an additional possible mechanism underlying neutrophilic airway inflammation regulated by FGF2. Notably, the association between FGF2 and neutrophils has been indicated in an IAV infection model as reported by Wang et al. [9]. In their report, FGF2 was induced by IAV infection and recruited neutrophils through an FGFR-dependent pathway. While we did not exclude the possibility of a direct role of FGF2 on neutrophils in this study, we did provide another possible mechanism by which FGF2 recruits subepithelial neutrophils via AECs.

Airway epithelial dysfunction and a number of airway epithelial-secreted inflammatory mediators have been characterised in neutrophilic asthma, including IL-1β, IL-6, and the well-recognised neutrophil chemoattractant IL-8 [33]. These cytokines have been found to be increased in the sputum or BALF of patients with neutrophilic asthma, be associated with disease severity, and predict uncontrolled asthma conditions in different cohort studies [34–36]. More importantly, mechanistic studies strongly suggest that these cytokines are potent inflammatory regulators in asthma, rather than only inflammatory biomarkers. Their expression levels are positively correlated with neutrophil percentage in induced sputum [37, 38]. Kim et al. [39] showed that intranasal administration of IL-1β alone to naïve mice could induce neutrophil responses, leading to cardinal features of severe, steroid-resistant allergic airway disease. Although this study did not provide detailed cellular mechanisms, we believe that IL-1β-mediated inflammatory responses in AECs play a crucial role. As further supporting evidence, a recent study conducted by Tan et al. [40] demonstrated that compared with a typical eosinophilic asthma model induced by low doses of HDM sensitisation and challenge, high doses of HDM intranasal administration to BALB/c mice induced neutrophilic airway inflammation, which is featured by increased IL-1β-mediated signalling in AECs. In our study, HDM stimulation upregulated FGF2 expression levels in both AECs and the asthma mouse model, and FGF2 pre-treatment recruited subepithelial neutrophils in vivo and promoted IL-1β-induced IL-6 and IL-8 secretion in vitro. This outcome provides an additional possible mechanism of airway epithelial-mediated neutrophilic airway inflammation modulated by the cooperation of FGF2 and the IL-1β signalling axis, warranting further investigation into the interaction of neutrophils and AECs regulated by FGF2. Moreover, intranasal administration of budesonide, a clinical medicine to treat asthma, was unable to suppress FGF2 overexpression in HDM-induced asthmatic mouse lungs, which might explain the clinical evidence of the correlation between FGF2 expression levels and asthma severity [6]. Further, the upregulation of FGF2 induced by allergens and its resistance to steroid treatments might be implied in other conditions such as uncontrolled allergic rhinitis that has been linked to asthma exacerbation [41], which is worthy of future study in this context.

In the current study, the activation of MAPKs, specifically ERK and p38MAPK, is associated with the synergistic effects of FGF2 and IL-1β on NF-κB activation, which is FGFR-dependent, as verified by an FGFR-specific inhibitor. This indicates that FGF2 functions as an inflammatory promoter, at least partly, if not all, through FGFR/MAPK/NF-κB signalling. It is worth noting that the regulation of inflammation by MAPKs not only exists in structural cells but also in immune cells, for which MAPKs promote cell responses and regulate immune cell differentiation and survival in the presence of inflammatory mediators or pathogens [42, 43]. Although this possibility in immune cells in asthma was not explored, co-localisation of FGF2 in airway or vascular infiltrated inflammatory cells was observed. Therefore, whether FGF2 plays a role in these cells through MAPK activation needs to be investigated.

It should be noted that our study outcome is contradictory to that of an earlier report that utilised an OVA-induced acute mouse model and administered FGF2 subcutaneously [10]. Indeed, both OVA- and HDM-induced models have widely been applied as powerful tools to study asthma pathogenesis in the last few decades. However, this may also lead to conflicting conclusions based on the intrinsic properties of allergens, and the OVA-induced asthma model has been questioned in recent years [44]. Compared with the OVA-induced acute mouse model, the HDM-induced chronic asthma model has several advantages. First, HDM, identified as one of the major allergens, is more clinically relevant, whereas OVA is not a certified human allergen. Accordingly, HDM-mediated pathophysiological pathways, such as airway epithelial barrier dysfunction and airway epithelial-driven inflammation, can better mimic clinical conditions and provide more consistent results in vivo and in vitro [45]. Second, the HDM-induced chronic asthma model applied in our study reproduces persistent asthma features, including chronic airway inflammation and airway wall remodelling, which can persist for a long time after the cessation of the HDM challenge (data not shown). In contrast, the OVA-induced acute model lacks chronic asthma features, and many acute features are short-lived [46]. Considering the potential role of FGF2 in both airway wall remodelling and immune function, we believe that our model is more applicable for this purpose. Moreover, another difference between these two studies is that in the OVA-induced acute mouse model, FGF2 was administered subcutaneously at a dosage that was 100 to 200 times higher than our dosage. In contrast, in our study, FGF2 was applied intranasally because we hypothesised that FGF2 might promote AEC-driven inflammation. The different routes of FGF2 administration might partly explain the contradictory results, which need to be further investigated.

Our study had several limitations. First, because of the difficulties and ethical issues associated with obtaining lung samples from asthma and healthy subjects, non-tumour samples collected from lobectomy were included, followed by a comparison between asthmatic and non-asthmatic subjects. Therefore, some of our observations may have been biased. Nevertheless, our observations are consistent with previous reports or the results of our mouse model [15]; thus, we believe that the impact of cancer in our study is minimal. Second, since all patients who underwent surgery passed the lung function test, patients with asthma with a lower lung function were not included. Moreover, all asthma patients enrolled in our study had received steroids for asthma treatment. Therefore, it is unclear whether FGF2 expression is associated with lung function parameters or steroid responses. Third, A549 cells were used as a cell model for AECs, which might conceal the functional differences in bronchial and alveolar epithelial cells [47]. Finally, the role of FGF2 in regulating immune cells [48–50] and other airway resident cells, such as ASMs and fibroblasts, which are critical players in modulating airway inflammation in asthma, were not investigated. These are the focus of our future research directions.

## Conclusions

This study revealed that FGF2 is overexpressed in both bronchial and alveolar areas in asthma patients, localised in AECs, subepithelial basement membrane, and inflammatory cell populations. By studying the HDM-induced chronic mouse model, it was demonstrated that FGF2 acts as an inflammation amplifier to promote airway inflammatory cell infiltration and recruit subepithelial neutrophils. This could partly be explained by an FGFR/MAPK/NF-κB-mediated pathway in AECs, leading to hypersecretion of inflammatory mediators. The outcome of this study provides evidence for further investigations into alternative or supplementary treatments for asthma.

## Supplementary Information

**Additional file 1: Table S1**. Clinical characteristics of patients with and without asthma.**Table S2**. Real-time quantitative PCR primer sequences of mouse and human genes.

**Addition file 2: Fig. S1**. Budesonide treatment did not suppress the upregulation of FGF2 protein abundance in the HDM-induced acute asthma mouse model. **a** Protocol of HDM-sensitised and HDM challenged acute mouse asthma model (scale bar = 50 μm). **b** Representative H&E images of lung sections among the groups. **c** Western blotting analysis showing FGF2 protein abundance among the groups. Data are represented as the mean ± standard error of the mean (n = 3). ^**^P < 0.01. Alum aluminium hydroxide, HDM house dust mite, i.p intraperitoneal.

## Data Availability

The data and materials used in the current study are available from the corresponding author upon request.

## Abbreviations

AECs: Airway epithelial cells
AEC II: Alveolar type II epithelial cells
ASMs: Airway smooth muscle cells
BALF: Bronchoalveolar lavage fluid
bFGF: Basic fibroblast growth factor
BSA: Bovine serum albumin
DMEM: Dulbecco’s modified Eagle’s medium
ELISA: Enzyme-linked immunosorbent assay
FGF2: Fibroblast growth factor 2
FGFRs: FGF receptors
HDM: House dust mite
H&E: Hematoxylin and eosin
HMW: High molecular weight
IAV: Influenza A virus
IL: Interleukin
LMW: Low molecular weight
OVA: Ovalbumin
pro-SPC: Pro-surfactant protein C
rh-FGF2: Recombinant human FGF2
rm-FGF2: Recombinant mouse FGF2
RT-qPCR: Real-time quantitative RT-PCR
SEM: Standard error of the mean
