# Supplementary material for: FGF2 is overexpressed in asthma and promotes airway inflammation through the FGFR/MAPK/NF-κB pathway in airway epithelial cells
Source: Mil Med Res. 2022 Jan 29;9:7. doi: 10.1186/s40779-022-00366-3 (PMC8800304; doi:10.1186/s40779-022-00366-3)
Supplement: Supplementary file 1 — Additional file 1: Table S1. Clinical characteristics of patients with and without asthma.Table S2. Real-time quantitative PCR primer sequences of mouse and human genes. [file 40779_2022_366_MOESM1_ESM.pdf]

Table S1 Clinical characteristics of patients with and without asthma

**Item**	**Non-asthmatic (*n* = 5)**	**Asthmatic (*n* = 5)**
Sex (M/F)	2/3	2/3
Age (year, mean ± SEM)	51.5 ± 5.4	48.4 ± 9.4
Lung function[a] (mean ± SEM)		
FVC (L)	3.26 ± 0.14	2.87 ± 0.41
FVC%pred (%)	98.00 ± 6.01	103.80 ± 10.60
FEV_1 (L)	2.66 ± 0.11	2.07 ± 0.40
FEV_1%pred (%)	95.96 ± 5.33	88.15 ± 6.60
Steroid treatment [*n*(%)]	0	5(100)
Intravenous steroids	0	1(20)
Oral steroids	0	1(20)
Inhaled steroids	0	3(60)
Bronchodilators [*n*(%)]	0	5(100)

[a] Information for one asthma patient is missing. *FEV_1%pred* forced expiratory volume in one second in percentage of predicted; *FEV_1* forced expiratory volume in one second; *FVC%pred* forced vital capacity in percentage of predicted; *FVC* forced vital capacity

Table S2 Real-time quantitative PCR primer sequences of mouse and human genes

**Gene**	**Forward primer**	**Reverse primer**
Mouse genes		
Rpl13a	CCTATGACAAGAAAAAGCGG	CAGGTAAGCAAACTTTCTGG
IL-4	TCACATTGTCACTGCAAATC	CCTTCTCAGTTGTGTTCTTC
IL-6	ATCACCCAGAACCAGAAG	ATGCAAGCTGGAAAACTG
IL-10	ATTTGAATTCCCTGGGTGAGAAG	CACAGGGGAGAAATCGATGACA
IL-13	ATCACCCAGAACCAGAAG	ATGCAAGCTGGAAAACTG
FGF2	CACCAGGCCACTTCAAGGA	GATGGATGCGCAGGAAGAA
Human genes		
GAPDH	ACAGTTGCCATGTAGACC	TTTTTGGTTGAGCACAGG
IL-6	GCAGAAAAAGGCAAAGAATC	CTACATTTGCCGAAGAGC
IL-8	GTTTTTGAAGAAAACTGAG	TTTGCTTGAAGTTTCACTGG

IL interleukin; *FGF2* fibroblast growth factor 2
