# Supplementary material for: FGF2 is overexpressed in asthma and promotes airway inflammation through the FGFR/MAPK/NF-κB pathway in airway epithelial cells
Source: Mil Med Res. 2022 Jan 29;9:7. doi: 10.1186/s40779-022-00366-3 (PMC8800304; doi:10.1186/s40779-022-00366-3)
Supplement: Supplementary file 2 — Addition file 2: Fig. S1. Budesonide treatment did not suppress the upregulation of FGF2 protein abundance in the HDM-induced acute asthma mouse model. a Protocol of HDM-sensitised and HDM challenged acute mouse asthma model (scale bar = 50 μm). b Representative H&E images of lung sections among the groups. c Western blotting analysis showing FGF2 protein abundance among the groups. Data are represented as the mean ± standard error of the mean (n = 3). **P < 0.01. Alum aluminium hydroxide, HDM house dust mite, i.p intraperitoneal. [file 40779_2022_366_MOESM2_ESM.pdf]

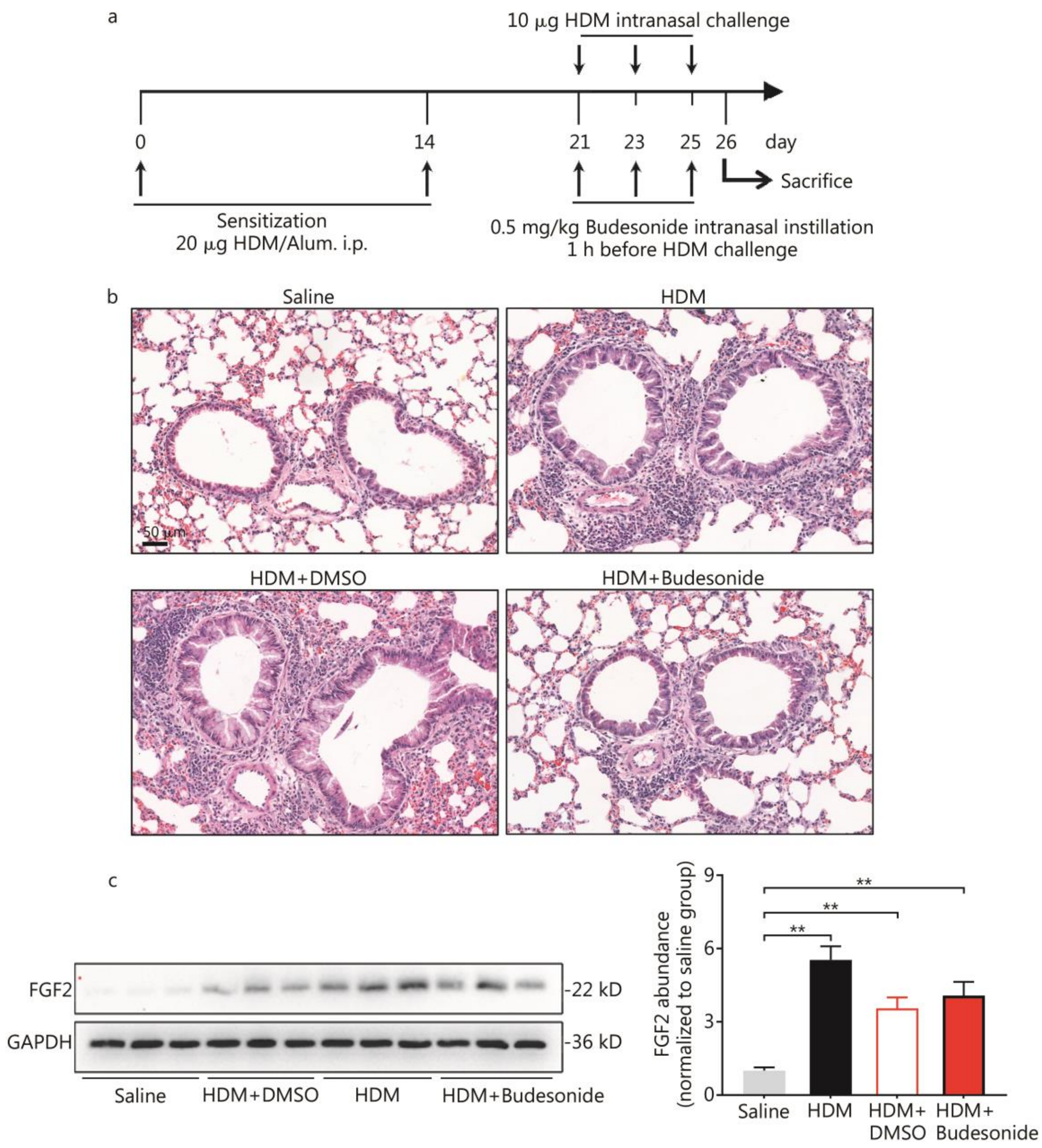

Fig. S1 Budesonide treatment did not suppress the upregulation of FGF2 protein abundance in the HDM-induced acute asthma mouse model.

a Protocol of HDM-sensitised and HDM-challenged acute mouse asthma model (scale bar = 50 μm). **b** Representative H&E images of lung sections among the groups. **c** Western blotting analysis showing FGF2 protein abundance among the groups. Data are represented as the mean ± standard error of the mean (n = 3). $^{**}P < 0.01$. Alum aluminium hydroxide, HDM house dust mite, i.p intraperitoneal
